# Etiology of diarrheal disease among children under 5 years in Egypt: a high incidence of human bocavirus

**DOI:** 10.1186/s42506-021-00084-z

**Published:** 2021-08-05

**Authors:** Neveen M. Rizk, Sherif Abd-Elmaksoud, Tarek M. Farid, Maha M. A. Abohashish, Ahmad Z. Al-Herrawy, Ibrahim A. Hamza

**Affiliations:** 1 Water Pollution Research Department, National Research Centre, 33 El Buhouth St., Dokki, Giza, 12622 Egypt; 2 Pediatric Department, National Research Centre, 33 El Buhouth St., Dokki, Giza, 12622 Egypt

**Keywords:** Human bocaviruses, Adenovirus, Rotavirus, Co-infections, diarrhea, qPCR

## Abstract

**Background:**

Human bocavirus (HBoV) is globally distributed and associated with respiratory and enteric infections. Limited data are available about the incidence of HBoV in Egyptian children. We aimed to investigate the association of HBoV genotypes in children with diarrheal disease and also to determine the possibility of HBoV co-infections with other human enteric pathogens.

**Methods:**

A total of 102 stool samples were collected from children under five years old with diarrhea. Samples were analyzed for the presence of HBoV by real-time PCR. HBoV positive samples were tested for adenovirus (AdV), rotavirus (RoV), parasitic helminths, and enteric protozoa.

**Results:**

HBoV was detected in 58% of examined cases. HBoV-3 was the most prevalent genotype observed (44%; 45 of 102), followed by HBoV-2/4 (33%; 34 of 102) and HBoV-1 (30%; 31 of 102). Although the incidence of HBoV was higher in males (66.6%; 34 of 51) than females (49%; 25 of 51), the analysis showed no significant difference for HBoV between genders. The average HBoV concentrations were 5.3 × 10^4^ GC/g in males and 1.03 × 10^5^ GC/g in females. Among the HBoV-positive samples, the single infection of HBoV was 52.5% (31/59), while the co-infections with multiple viruses were found in 1.7% (1/59) for HBoV and AdV, 33.9% (20/59) for HBoV and RoV, and 11.9% (7/59) for HBoV, and RoV and AdV. No co-infection with parasitic helminths or enteric protozoa was found.

**Conclusions:**

The single infection of HBoV in some children suffering from acute gastroenteritis indicated that HBoV could be the main etiologic agent of the disease. The study highlights the high incidence of HBoVs genotypes with remarkable multiple co-infections in the pre-school children in Egypt.

## Introduction

Diarrheal disease is the second most common cause of mortality worldwide in children less than 5 years and it is estimated that 600,000–700,000 infants and young children die from diarrhea each year. Most of the deaths occur in Sub-Saharan Africa and South Asia and mortality is high in children less than 5 years [1]. Mortality is uncommon in high-income countries, but diarrhea is often associated with substantial medical and healthcare costs. Viral diarrhea is  a prevalent type of diarrhea in the world affecting patients of all ages, especially children [1]. Enteric viruses are the most frequent common pathogens causing diarrhea in high-income as well as low-income countries [2]. The most common agents are rotavirus (RoV), adenovirus (AdV), norovirus (NoV), sapovirus (SaV), and astrovirus (AstV). Additionally, bocaviruses (HBoVs) are increasingly being identified as causative agents of diarrhea [3]. However, little attention for HBoVs as a causative agent of diarrhea has been received so far, particularly the co-infections pattern of different HBoVs genotypes in pre-school children.

Human bocavirus (HBoV) was discovered in 2005 [4]. HBoV is a member of the family Parvoviridae, subfamily Parvovirinae, genus Bocaparvovirus. Four HBoV genotypes (HBoV-1-HBoV-4) have been identified and characterized by a 5.3 kb single-stranded DNA genome, encapsulated in a non-enveloped icosahedral capsid protein coat [5]. The genome of HBoV has three open reading frames (ORFs), encoding two nonstructural proteins NS1 and NP1 and two viral capsid proteins VP1 and VP2 [6].

A plethora of studies detected HBoVs in Europe [7], North America [8], South America [9], the Middle East [10], Asia [11], and Australia [12]. In African countries, HBoVs have been reported in Egypt 2% (2/100 children of age from 1 month to 2 years) [13], Kenya 1.8% (7/384 children/adults) [14], and South Africa 22.8% (174/1460 children < 2 years) [15]. As yet, little is still known about the prevalence of HBoV and its genotypes in Egypt, particularly in children.

HBoVs cause a variety of clinical manifestation and could be isolated from several types of clinical samples including nasopharyngeal aspirates [12, 16] and stool [17]. Furthermore, HBoVs was also found in environmental waters [18, 19]. HBoV-1 is commonly associated with respiratory tract infections in pediatric patients, as well as in those with gastrointestinal symptoms [20]. In contrast, the other three genotypes (HBoV2-4) are found mainly in stool samples from patients suffering from gastroenteritis [21].

The association of HBoV with respiratory disease and acute gastroenteritis remains unclear due to the high rate of co-infection with other pathogenic viruses in symptomatic patients, as well as its frequent detection in asymptomatic individuals [6, 21]. However, HBoV was isolated from hospitalized infants suffering from respiratory infections, without other respiratory viruses (i.e., adenovirus, respiratory syncytial virus, parainfluenza virus 1, 2, and 3, human metapneumovirus, influenza virus A and B) [22]. Moreover, some reports found single infection of HBoV in the stool of infected patients with acute gastroenteritis (AGE), particularly among children under 5 years of age [3, 23]. However, all children of different ages are at risk of HBoV infection  as a result of poor hygiene practices and sanitation [23]. It is suspected that HBoV enters the bloodstream after a long period of persistence in the mucosa of the respiratory tract and migrates to the gastrointestinal tract, where it may either produce new infection or be excreted asymptomatically [24]. HBoVs can infect all human age groups, although severe infections were noticed in children [25] and patients with underlying diseases like cancer [26]. In this study, we specifically aimed to investigate the association of HBoV genotypes in children with AGE up to 5 years. Also, we screened the positive HBoV samples for AdV, RoV, parasitic helminths, and enteric protozoa to determine whether the causative agent of diarrhea in the collected samples is HBoV or co-infection with other pathogens.

## Methods

### Sample collection

A total of 102 stool samples were collected anonymously from preschool children (51 samples from males and 51 samples from females) from private clinics in Giza, Egypt. The samples were collected from children under 5 years of age suffering from acute watery diarrhea. Stool samples were examined immediately at the same day for parasites identification. In order to avoid repeated freezing and thawing, stool samples were aliquoted, stored at − 20 °C, and tested within a month from collection for HBoV.

### Sample concentration and processing

About 100 mg of fecal diarrhea samples were weighed and diluted in phosphate buffer saline (1:10). The samples were vortexed for 30 s followed by centrifugation at 5000 rpm for 10 min at room temperature. The supernatants were kept at − 80 °C until further use.

### Nucleic acid extraction

Viral nucleic acids were extracted from 200 μl of the concentrated sample using GeneJET Viral DNA and RNA Purification kit (Thermo Scientific-USA) according to the manufacturer’s instructions. The obtained nucleic acid was dissolved in 60 μl of eluent and kept at – 80 °C until use.

### Detection and quantification of HBoV by qPCR

All primers used in the current study were listed in Table 1. The quantification protocol targeting the NP1 gene for HBoV-1 was used according to Hamza et al. [18]. A single sense primer was shared in HBoV-2, 3, and 4 quantifications and the qPCR of HBoV-2 and 4 used the same antisense primer [29]. SYBR green qPCR assay was conducted for HBoVs quantification using a Maxima SYBR Green qPCR Master Mix Kit (Thermo Scientifc). The PCR conditions were 10 min initial denaturation step at 95 °C, 40 cycles of denaturation at 95 °C for 15 s and annealing-extension at 60 °C for 1 min. Amplification was followed by one cycle of melting curve analysis. Dissociation was carried out from 60 °C to 95 °C with a temperature ramp of 0.05 °C/s. Analysis indicated a melting peak (Tm) at 83 °C ± 0.2 °C for HBoV-1, 81.5 °C ± 0.3 °C for HBoV 2/4, and 80 °C ± 0.2 °C for HBoV-3. PCR amplification and data analysis were performed by CFX 96 Realtime PCR machine (Bio-Rad). The genome copy numbers of bocavirus genotypes (HBoV-1, HBoV-2/4, and HBoV-3) were determined by comparison with a standard curve generated with serial dilutions of positive control of the PCR product from each genotype. The PCR product was purified using Wizard® SV Gel and the PCR Clean-Up System (Promega, USA). Nucleic acid concentrations of the purified PCR products were determined by NanoDrop Fluorospectrometer (Thermo-Scientific, USA). The DNA concentration was converted to genomic copies using the following formula: number of DNA copies = (DNA amount (ng) × 6.022 × 10^23^)/(length (bp) × 1 × 10^9^ × 650).

**Table 1:** Primer sequences of HBoVs, AdV, and RoV

Virus	Target gene	Primer name	Sequence (5′–3′)	Fragment length (bp)	References
**HBoV-1**	NP1	NP1-F2421	TGGCAGACAACTCATCACAG	123	[22]
		NP1- R2544	TCTTCGAAGCAGTGCAAGAC		
**HBoV-2/4**	NS1	HBoV234F	GCACTTCCGCATYTCGTCAG	100	[29]
		HBoV24R	AGCAGAAAAGGCCATAGTGTCA		
**HBoV-3**	NS1	HBoV234F	GCACTTCCGCATYTCGTCAG	100	
		HBoV3R	GTGGATTGAAAGCCATAATTTGA		
**RoV**	VP6	VP6-F	GACGGVGCRACTACATGGT	382	[40]
		VP6-R	GTCCAATTCATNCCTGGTG		
		VP6-NF	GCTAGAAATTTTGATACA	147	
		VP6-NR	TCTGCAGTTTGTGAATC		
**AdV**	Hexon	HexAA1885	GCCGCAGTGGTCTTACATGCACATC	300	[31]
		Hex1913	CAGCACGCCGCGGATGTCAAAGT		

The standard curve of each bocavirus was separately prepared by tenfold serial dilution of the nucleic acid standard ranging from 5 × 10^1^ to 5 × 10^7^ copies/reaction. For HBoV-1 and HBoV-3, the slop was − 3.69; the coefficient of determination (R^2^) was 0.99. In the case of HBoV-2/4, the mean value of the slope was − 3.45; the mean of R^2^ was 0.99. Virus concentration per gram (g) GC/g was calculated according to the following equation: *GC*/*g* = *GCxDF*× 10, where GC is genome copy number per reaction, DF is the dilution factor for the volume reductions that occur during the concentration, DNA extraction, and qPCR steps, and the obtained GC was expressed per gram of stool sample.

### Detection of rotavirus and adenovirus

The positive samples for HBoV were tested for RoV and AdV to determine the pathogen co-infections. RoV was detected by using nested RT-PCR for the detection of the VP6 segment [30]. AdV was detected according to Puig et al. [31] using primers based on the hexon gene (Table 1).

### Detection of enteric protozoa and helminth parasites

The positive samples for HBoV were tested for enteric protozoa and helminth parasites by microscopic examination (direct wet-mount preparation) [32] to differentiate whether AGE originated from HBoV or other causative agents.

### Statistical analyses

Statistical analyses were performed using GraphPad Prism version 8.3.0 software (USA). The critical *P* value for the test was set at < 0.05. One-way ANOVA shows the significant difference between the relative distribution of different bocavirus genotypes in total, male and female stool samples. The unpaired *t* test was used to compare each HBoV genotype in male versus female stool samples.

## Results

### Detection and quantification of human bocaviruses

In the present study, human bocavirus genotypes were detected in 58% (59 of 102, *P <* 0.05) of the children stool samples using qPCR, which targets NP-1 and NS-1 genes. Statistically, the viral type showed a significant effect on the prevalence of HBoV in males (*P* = < 0.0001), although no significant influence on the prevalence of the virus in females (*P* = 0.27). The detection rates of different HBoV genotypes are presented in Table 2.

**Table 2:** Detection rates of bocaviruses genotypes in stool samples of under five children, Giza, Egypt

	Prevalence n/N (%)		
	Male	Female	Total
**Bocavirus infection**			
HBoV-1	19/51 (37.3%)	12/51 (23.5%)	31/102 (30.4%)
HBoV-2/4	20/51 (39.2 %)	14/51 (27.5%)	34/102 (33.3%)
HBoV-3	25/51 (49 %)	20/51(39.2%)	45/102 (44.1%)
**One genotype** (*n* = 20/59, 34%)			
HBoV-1	2/34 (5.9%)	1/25 (4%)	3/59 (5%)
HBoV-2/4	4/34 (11.8%)	3/25(12%)	7/59 (12%)
HBoV-3	5/34 (14.7%)	5/25(20%)	10/59 (17%)
**Two genotypes** (*n* = 27/59, 46%)			
HBoV-1 + HBoV-2/4	3/34 (8.8%)	1/25 (4%)	4/59 (6.7%)
HBoV-1 + HBoV-3	7/34 (20.6%)	5/25 (20%)	12/59 (20.3%)
HBoV-2/4 + HBoV-3	6/34 (17.6%)	5/25 (20%)	11/59 (18.6%)
**Three genotypes** (*n* = 12/59, 20%)			
HBoV-1 + HBoV-2/4+ HBoV-3	7/34 (20.6%)	5/25 (20%)	12/59 (20%)

*n* number of bocavirus positive cases, *N* total number of cases

### HBoV-1

**Fig. 1:**
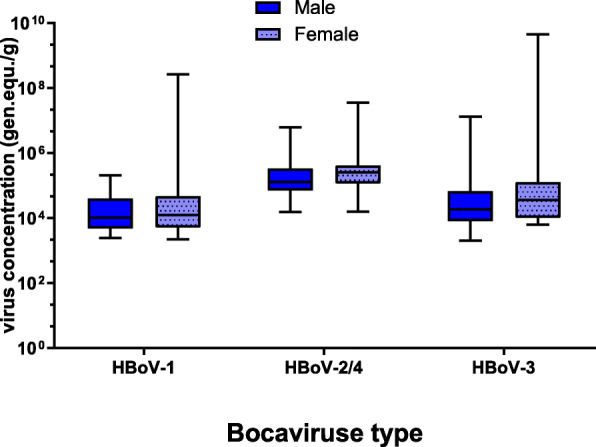
Concentration of bocaviruses in male and female stool samples of under five children

HBoV-1 was detected in 31 out of 102 stool samples. The positive samples were 37% from males and 23% from females, in accordance with the number of samples collected from each gender (Table 2). Statistically, there was no significant difference between male and female patients who were infected with HBoV-1 (*P* = 0.22). In other words, gender had no significant effect on the prevalence of HBoV-1. The average viral concentration was 1.0 × 10^4^ GC/g in males and 1.3 × 10^4^ GC/g in females (Fig. 1).

### HBoV-2/4

HBoV-2/4 virus was detected in 34 out of 102 samples. The prevalence of HBoV-2/4 was higher in males (39.2 %) than females (27.5%) (Table 2). Similar to HBoV-1, no critical role played by the gender on the prevalence of HBoV-2/4 (*P* = 0.4254). The average viral concentrations were 1.3 × 10^5^ GC/g and 2.6 × 10^5^ GC/g in males and females, respectively (Fig. 1).

### HBoV-3

HBoV-3 showed a high prevalence (44.1%) in total samples compared to other HBoV types. The prevalence of HBoV-3 was higher in males (49 %) than females (39.2%) (Table 2). HBoV-3 had the same pattern as HBoV-1 as well as HBoV-2/4 in males versus females. The mean virus concentration in male and female samples reached 1.9 × 10^4^ and 3.6 × 10^4^ GC/g, respectively (Fig. 1).

### HBoV genotype co-infections

Among the positive samples, single genotypes detected in 34% (20/59) of HBoV positive cases and two or more genotypes detected in 66% (39/59) of HBoV positive cases. The most common mixed genotype cases were HBoV-1 and HBoV-3 (20.3%), followed by HBoV-2/4 and HBoV-3 (18.6%) and HBoV-1 andHBoV-2/4 (6.7%) (Table 2).

### HBoV and other enteric pathogen co-infections

**Fig. 2:**
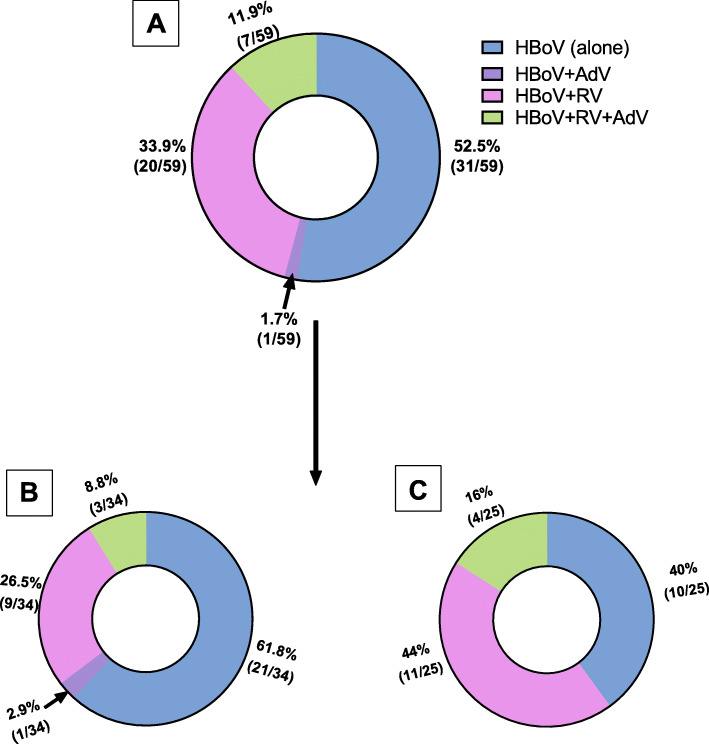
Different infection types in HBoV-positive samples. **A** Total positive samples for HBoV (*n* = 59). **B** Males positive samples. **C** females positive samples

The positive samples for HBoV (n = 59) were screened for other viruses (i.e., AdV and RoV), protozoa, and parasitic helminths to explore whether AGE originated from bocavirus or other causative agents. The single infections of HBoV in males and females were 61.8% (21/34) and 40% (10/25), respectively, while the co-infections ratios of HBoV/AdV, HBoV/RoV, and HBoV/RoV/AdV in males were 2.9 % (1/34), 26.5% (9/34), and 8.8% (3/34), respectively. In females, multiple infections of HBoV/RoV and HBoV/RoV/AdV account for 44% (11/25) and 16% (4/25), respectively (Fig. 2). Protozoa and parasitic helminths could not be detected in fecal samples of the children suffering from viral infections.

## Discussion

Different viruses including RoV, AdV, NoV, and AstV are the major cause of gastrointestinal disease worldwide, particularly in developing countries. Globally, HBoV has been associated with about 5.9% of gastrointestinal illnesses and 6.3% of respiratory tract infections [6, 12] and has been reported in various studies as a potential cause of diarrhea outbreaks [33]. It was estimated that 13% of African individuals suffering from gastroenteritis principally caused by HBoVs between 2005 and 2016 [23]. To our knowledge, limited data are available about the incidence of HBoV in Egyptian children. Only two clinical studies were reported in Egypt; the first study found HBoV in Children with AGE [13]. The second study observed HBoV-1 in children suffering from lower respiratory tract infections without providing any data about different genotypes in the given cases [27]. However, HBoV-1, HBoV-2, and HBoV-3 were detected in environmental samples from Egypt [19]. Therefore, it is crucial to determine the prevalence of HBoV genotypes in children < 5 years of age, who are most vulnerable to HBoV infections.

Our results showed that the proportion of the single infection of HBoV (52.5%, 31/59) was higher than co-infection with AdV and RoV, indicating the possible contribution of the virus in the pathogenicity. In contrast, infection with HBoV in Pakistani children was not significantly associated with gastroenteritis alone where 98% of HBoV reported cases had co-infection with RoV [34]. The co-infection of HBoV and RoV (46%) in patients with gastrointestinal infections has been recorded elsewhere [6]. In this study, mixed infections with the three viruses (HBoV, AdV and RoV) were detected in 11.9% of HBoV-positive samples. Likewise, the mixed infections with (Aichivirus, sapovirus, human parechoviruses, bocavirus, and rotaviruses) were detected in 45.4% of the stool samples from Indian children < 5 years of age hospitalized for acute gastroenteritis [28].

In the present study, the prevalence of HBoV in children suffering from AGE was 58% which is higher than the previous reports of AGE associated with HBoV in Egypt (2%) [13], Brazil (24 and 42%) [35], and Taiwan (8.5%) [36]. This difference in detection rate could be attributed to the difference of sensitivity of the detection method, geographical region, hygiene and sanitary conditions, and/or the sample size of the study. HBoV has been detected in stool samples of both children and adults; however, children ≤ 2 years of age were found to be most susceptible to HBoV infection [23]. In our study, we detected HBoV-1, HBoV2/4, and HBoV-3. Likewise, all genotypes have been detected in USA (children), Finland (children/adults), Japan (children), Kenya (children/adults), and Turkey (children) [23]. The most abundant HBoV type in the current study was HBoV3. In contrast, HBoV1 was the most prevalent in urban and rural settings followed by HBoV-2 [12, 23, 34]. The differences in HBoV genotypes abundance may be due to regional differences in viral epidemiology or because not all the studies tested all HBoV genotypes or the lately discovered genotypes (i.e., HBoV-2/4 and HBoV-3) compared to HBoV-1. HBoV was found in 9 % of nasopharyngeal swabs obtained from children with acute respiratory tract infection in Alexandria, Egypt [37]. The HBoV-1 was the only genotype detected, suggesting that a single genetic lineage of HBoV is circulating in Egypt [37]. However, in the present study, HBoV-3 was the most abundant genotype which could be due to the difference in samples types. Furthermore, the relative abundance of HBoV-2 and HBoV-3 compared to HBoV-1 may be due to differences in tissue tropism or pathogenesis among HBoV genotypes, which may affect transmission and persistence.

In the present study, the viral incidence was higher in males 66.6% than females 49% and the difference was statistically non-significant (*p* > 0.05). The current results agree with the study of Nawaz et al. [38] from the UK, who reported that the distribution of HBoV among females and males was not significantly different, which recorded 53% and 47% in females and males, respectively. Similarly in Brazil, the researchers found that 57% of HBoV positive cases were detected in boys and 43% were detected in girls [39]. In another study in Pakistan, the researchers found HBoV infection rates were higher in males (68%) as compared to females (32%) [34].

### Limitations of the study

Our study had some limitations such as the lack of  a healthy control group and screening of other enteric pathogens (e.g., bacteria, sapovirus, norovirus, or astrovirus).

## Conclusions

The presence of HBoV in some children suffering from AGE without the association of any other etiological agents (i.e., AdV, RoV, protozoa and parasitic helminths) indicates the ability of the virus to cause the disease. HBoV was abundant in stool samples from children with gastrointestinal disease in Egypt. Higher infection rates were detected in males rather than females. However, the viral loads were higher in females than in males. HBoV-3 was the most abundant among HBoV genotypes. Higher proportions of multiple co-infection of HBoV genotypes were recorded compared to single infections. The viral type had a strong significant effect on the prevalence of HBoV rather than human gender. Moreover, the concentration of HBoV-2/4 was higher than HBoV-1 as well as HBoV-3. Taken together, the data obtained in our study raise a concern on the role of HBoV in gastrointestinal illness.

## Data Availability

The datasets used and analyzed during the current study are available from the corresponding author upon reasonable request.

## Abbreviations

ANOVA: Analysis of variance
HBoV: Human bocavirus
AGE: Acute gastroenteritis
AdV: Adenovirus
RoV: Rotavirus
NoV: Norovirus
SaV: Sapovirus
AstV: Astrovirus
GC/g: Gene copy per gram
RPM: Revolutions per minute
n: Number
*P*: P value
RT-PCR: Reverse transcription polymerase chain reaction
DF: Dilution factor
ORFs: Open reading frames
qPCR: Real-time PCR
R^2^: The coefficient of determination
UK: United Kingdom
USA: United States of America
